# Effect of Income Level and Perception of Susceptibility and Severity of COVID-19 on Stay-at-Home Preventive Behavior in a Group of Older Adults in Mexico City

**DOI:** 10.3390/ijerph17207418

**Published:** 2020-10-12

**Authors:** Maria Esther Irigoyen-Camacho, Maria Consuelo Velazquez-Alva, Marco Antonio Zepeda-Zepeda, Maria Fernanda Cabrer-Rosales, Irina Lazarevich, Antonio Castaño-Seiquer

**Affiliations:** 1 Health Care Department, Metropolitan Autonomous University, Unit Xochimilco, Mexico City 04960, Mexico; meirigo@correo.xoc.uam.mx (M.E.I.-C.); mzepeda@correo.xoc.uam.mx (M.A.Z.-Z.); mcabrer@correo.xoc.uam.mx (M.F.C.-R.); iboris@correo.xoc.uam.mx (I.L.); 2 Director of The Master Program of Family and Community Dentistry, Faculty of Dentistry, University of Sevilla, Street Avicenna, 41009 Sevilla, Spain; acastano@us.es

**Keywords:** COVID-19, older adults, SARS-CoV-2, income level, educational status, quarantine, Mexico

## Abstract

Early information on public health behaviors adopted to prevent the spread of coronavirus (COVID-19) may be useful in controlling the Severe Acute Respiratory Syndrome Coronavirus 2 (SARS-CoV-2) transmission. The objective of this study was to assess the role of income level (IL) and the perception of older adults, regarding COVID-19 susceptibility and severity, on adopting stay-at-home preventive behavior during the first week of the outbreak in Mexico. Participants in this cross-sectional study were urban community dwellers, aged ≥ 65 years from Mexico City. A total of 380 interviews were conducted over the phone. The mean respondent age was 72.9 years, and 76.1% were women. Over half (54.2%) of the participants perceived their susceptibility to COVID-19 as very low or low. Similarly, 33.4% perceived COVID-19 severity as being very low or low, and 57.6% had decided to stay at home: this behavior was associated with IL (β = 1.05, *p* < 0.001), and its total effect was partially mediated (15.1%) by perceived severity. Educational attainment was also associated with staying at home (β = 0.10, *p* = 0.018) and its total effect was partially mediated (15.0%) by perceived susceptibility. Interventions aimed at low income and less educated older adults should be developed to improve preventive behaviors in this vulnerable group during the COVID-19 pandemic.

## 1. Introduction

The Severe Acute Respiratory Syndrome Coronavirus 2 (SARS-CoV-2) epidemic was first reported by the World Health Organization (WHO) Chinese Office on 31 December 2019. WHO called an international health emergency on 30 January and requested global collaboration to control the spread of the virus [1]. The epidemic rapidly spread to Asian and European countries. On 26 February 2020, SARS-CoV-2 was introduced to the United States and Latin American countries from China and Europe. As a result of the global spread of the disease, WHO raised the threat to “very high” on 28 February 2020 [2]. On that same date, Mexico confirmed its first coronavirus (COVID-19) case, a 35-year-old male who had spent time in Italy in the previous weeks [3]. The Mexican Ministry of Health provided standardized guidelines for epidemiological and laboratory surveillance for the detection of COVID-19 cases in early February [4].

By mid-March 2020, the pandemic was in its initial stages in several Latin American and African countries. To control the spread of this disease, it is essential to understand the factors that may contribute to the population-wide adoption of preventive behaviors. On 24 March, the Mexican government implemented the DN III Plan, which is operated by the Ministry of Defense to help civilians in cases of disaster. Army healthcare staff helped in the treatment of COVID-19 cases in the civilian population, and provided the logistics required to mitigate the epidemic. Phase 2 of the epidemic was also established on 24 March, as a result of the identification of local transmission of the virus. The measures taken by the government included a prohibition on meetings, and the closure of malls, gyms, churches, bars, and other places where social gatherings take place, to avoid transmission. However, epidemic control is primarily based on self-isolation. Preventive practices, such as hand washing and the disinfection of surfaces, have also been widely advised. Thus, cooperation of the population is crucial to reduce the number of new cases [5].

Mexico has more than 120 million inhabitants; with approximately 15.4 million of those aged 60 years or older [6]. The pandemic affects all age groups; however, the case-fatality rate is higher among people 65 years and older [7]. Therefore, it is important to investigate the knowledge, perception and practices of older people to develop adequate and rapid strategies to contain the epidemic.

There is a socioeconomic gradient for morbidity and mortality rates and a strong association between low income and poor health, with lower socioeconomic groups carrying a higher burden of disease [8,9]. Socioeconomic position (SEP) is a complex construct, which includes several factors, such as household income, household conditions, occupation, education, and unemployment [10]. SEP is also a risk marker for infectious disease outbreaks; different pathways intervene to increase the probability of contagion in disadvantaged groups [11]. Consequently, in relation to COVID-19, SEP has been suggested to have an effect on behavior that correspondingly affects morbidity and mortality [12]. Income level (IL), as an indicator of SEP, is associated with different health outcomes [13]. This indicator is considered as one of the best estimates of material living standards [10,14]. In Mexico, adults aged ≥65 years receive a universal pension from the government, of $1275 Mexican pesos (approximately $51 US dollars) per month [15]; in 2019, approximately 8 million people received this compensation. The benefits of this payment to older adults, in a variety of factors, such as health status and well-being, have been demonstrated [16,17].

Physical distancing, the use of face masks, hand washing, covering the mouth and nose when coughing or sneezing, and staying at home are among the behaviors proposed by WHO to reduce the spread of SARS-CoV-2 [18]. Since the beginning of the epidemic, it has been critical to adopt these behaviors, particularly staying at home, which has been shown to reduce the risk of spread in several countries [19]. However, it is not currently well understood how the perception of susceptibility and the severity of COVID-19 influences the adoption of preventive behaviors by older adults.

The Health Belief Model (HBM), developed from the general framework of Social Cognitive Theory, has been used to better understand the process of preventive behavior adoption by older adults [20,21]. Mexico is currently in the process of controlling the virus spread, and the behavior of citizens is crucial to controlling transmission. It is the government’s duty to provide norms and protocols to control the epidemic, and the community’s responsibility to comply with them [22]. Thus, the knowledge and perception of individuals may lie along the pathway leading to the adoption of preventive health behaviors. With infectious diseases, increased knowledge has been associated with better adherence to treatment [23]: a study in China found that going to crowded places and not wearing a face mask was associated with poor knowledge of COVID-19 [24]. This finding is particularly important for older adults, given their high risk of mortality [7].

Therefore, this study aimed to assess how socioeconomic status, as determined by IL, and the perception of susceptibility and severity of COVID-19 by older adults affected their adoption of stay-at-home preventive behavior, at the beginning of the outbreak in Mexico.

## 2. Materials and Methods

### 2.1. Design and Participants

A cross sectional study was undertaken, using a structured questionnaire administered by phone interviews in Mexico City between 13 March and 21 March 2020. The study participants were recruited from another study that had recently begun evaluating aging, nutrition, and physical performance in the older residents of Mexico City. Participants in the cohort were recruited from a recreational center in the south-eastern area of Mexico City that holds actives for senior citizens. All participants of the initial study were asked to participate in the COVID-19 survey. The participants were 65 years or older and urban community dwellers. Exclusion criteria included individuals unable to ambulate by themselves, and those with a psychological or physical condition that did not allow them to answer the questionnaire. The study was approved by the Ethics Committee of the Metropolitan Autonomous University-Xochimilco (Division of Biological and Health Sciences, DCBS/52-17-20), and the modifications related to COVID-19 sub-study were also registered. Participants were asked to answer a yes-no question to confirm their willingness to voluntarily participate in the COVID-19 phone survey.

A sample size calculation for testing odds ratio (OR) equality was performed to determine whether the OR between two groups is different to the null value (OR = 1). The sample size of 356 participants allows for a type I error of 0.05 and a power of 0.80, a proportion of low IL participants who had the outcome “stay at home” of 0.20 and a proportion of middle-income participants with the outcome “stay at home” of 0.35 [25].

A total of 417 individuals were asked to participate in the study, this is the total number recruited from the initial study. The response rate was 91.13%: those that did not answer the questions cited lack of time or unwillingness to participate. One individual was ill, and one died before the interview could be conducted. A total of 380 telephone interviews were completed. Two trained interviewers administered a six-item standardized questionnaire. The questions were “ad hoc”, because at the time of the study, no previous questionnaires for older adults and COVID-19 perception were available. The interviewers assured the respondents that any information given would be confidential, that their name was not linked to their responses, and that not answering questions would have no detrimental consequences. The interviewers were trained to remain neutral and maintain a formal relationship with the participants during the interview process. Face validity was assessed by three researchers with experience in health behavior evaluations, who reviewed the question and answer choices of the questionnaire [26]. A pilot study was conducted in 20 older adults to evaluate the instrument and make adjustments. Two changes were implemented after reviewing the results of the pilot study. One question was changed from: “Compared with other people, how likely are you to get SARS-CoV-2?” to: “Compared with other people, how likely are you to get COVID-19?” In question 6, that asked for the sources of COVID-19 information, the option “family and friends” was added (Table 1).

Participants’ knowledge of COVID-19 was assessed by asking for symptoms of the disease, and identifying the age group that experiences more complications as a consequence of COVID-19. In addition, the participants were asked about their main source of information about the pandemic, as well as the preventive behaviors they had adopted as a result.

Staying at home, as a preventive behavior, was the outcome variable. This was identified when participants indicated that, as a result of the epidemic, they only went out for basic needs, such as going to the supermarket or to the drug store. IL predictor variable was determined by the monthly household income. To classify IL, we applied the criteria of the National Institute of Statistic Geography and Informatics in Mexico (INEGI). INEGI provides information of per household income distribution by deciles: the lowest decile corresponds to a monthly income of $3038 Mexican pesos ($122 US dollars), the fifth decile is $10,773 ($431 US dollars), and the highest income decile corresponds to ≥$55,583 ($2224 US dollars), (exchange rate at 3 April 2020) [27]. The participants were asked about their income as follows: “Approximately, how much is the monthly income in your household?” Five ranges were provided as possible answers (in Mexican pesos): <$5000 ($200 US dollars), $5000–$10,000 ($200–$400 US dollars), $10,000–$15,000 ($400–$600 US dollars), $15,000–$20,000 ($600–$800 US dollars), and >$20,000 ($800 US dollars). Participants with an income lower than the fifth decile were considered as the low-income group, all others were placed in the middle IL group. There were no participants in the two highest income deciles. Two variables were considered as possible mediators: perceived susceptibility was measured by asking the participants “Compared to other people, how likely are you to get COVID-19?” and perceived severity was measured by asking “How severe do you think COVID-19 infection is?” (Table 1).

### 2.2. Statistical Analysis

The present analysis is based on 380 older adults, with no missing data for all the variables examined. A generalized structural equation model (GSEM) was used to develop the path and mediation analyses. GSEM omits drawing the covariance between the observed exogenous variables by default [28]. The Akaike information criterion (AIC), and the Bayesian information criterion (BNIC), were used in the model selection. Direct, indirect, and total effects were estimated using Bayesian estimates for 1000 Bootstrap repetitions, using the Buis technique for mediation evaluation [29]. Crude, adjusted ORs, and 95% confidence intervals (95% CI) were obtained. The *p*-value used for statistical significance was *p* < 0.05. Stata V.15 (Stat Corp., College Station, TX, USA) was used for the data analyses.

## 3. Results

A total of 380 adults participated in the study; the majority were female (76.1%), and the mean age was 72.9 years (±8.0) (Table 2). No difference in the mean age was found by sex (*p* > 0.05): the average age of the females was 72.9 years (±8.8), and 72.7 years (±7.8) for the males. Table 2 presents the sociodemographic characteristics of the participants. Less than half of the participants were in the low IL group (44.2%). Approximately one-fifth (19.5%) had less than three years of formal education, and a quarter (25.3%) had undertaken a bachelor’s degree, or had a higher degree, which implies more than ten years of formal schooling.

Knowledge about COVID-19 was assessed based on the identification of symptoms. Fever was identified by most participants (57.9%), followed by a cough (47.1%), while 46 (12.1%) participants were unable to describe any COVID-19 symptoms (Table 2). Most participants (76.3%) could describe three or more symptoms, while 11.6% could describe one or two symptoms. Approximately two-thirds (69.5%) correctly identified older adults as the high-risk age group.

Most of the participants (67.6%) stated that television was their source of COVID-19 information, while radio was mentioned by less than one-third of the participants. Friends and family were the information source for 15.8% of participants, and few respondents (11.6%) used the web or social media to inform themselves of the pandemic (Table 2).

**Figure 1:**
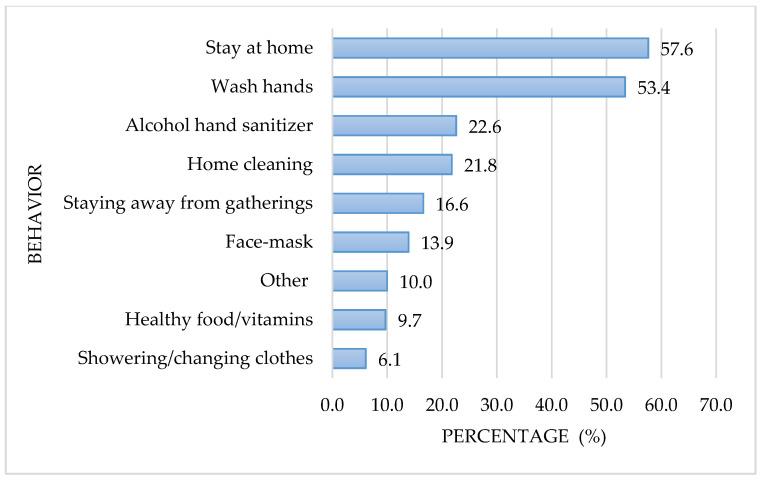
Frequency (%) of preventive behaviors associated with the coronavirus (COVID-19) pandemic in adults (≥65 years old) in Mexico City.

Figure 1 shows the preventive measures against COVID-19 adopted by older adults. The most frequent preventive measure was staying at home (57.6%), followed by hand washing (53.4%). The use of alcohol hand sanitizers and household cleaning and disinfection as preventive measures was adopted by 22.6% and 21.8%, respectively. Sixty-three (16.6%) of the participants had not taken any preventive measures against contagion, at the time of the interview.

Adoption of the stay-at-home preventive behavior for COVID-19 was analyzed by taking into consideration sociodemographic variables, together with the level of knowledge, and source of information regarding the disease (Table 3). No association was found between stay-at-home behavior and age (*p* = 0.875) or sex (*p* = 0.529). Middle IL participants were more than three times as likely to stay at home compared with the low IL group (OR = 3.40, *p* < 0.001). Similarly, higher educational attainment was associated with stay-at-home behavior: participants with more than 10 years of schooling were more likely to adopt this preventive behavior than those with incomplete elementary-level schooling (OR = 3.18, *p* < 0.001). Knowledge regarding the age group at higher risk of complications from COVID-19 (i.e., those answering “older adults”) were more likely to stay at home (OR = 1.56, *p* = 0.046). With the exception of fever (OR = 1.72, *p* = 0.010), knowledge of COVID-19 symptoms was not associated with the adoption of the stay-at-home preventive measure. Regarding COVID-19 information sources, only those who used the web or social media were more likely to adopt stay-at-home behavior (OR = 3.36, *p* < 0.001).

Table 4 presents the perception of older adults, regarding susceptibility to and severity of COVID-19. A perception of low susceptibility was reported by approximately half of the participants (43.4%). Both perception categories for the extremes of susceptibility (very high and very low risk) were identified by 10.8% of participants. No significant association was found between watching television as a source of COVID-19 information and perceived susceptibility (*p* = 0.087). Similarly, no association was found between perceived susceptibility and using the web and social media to obtain information (*p* = 0.261). However, susceptibility perception was associated with stay-at-home behavior (Table 4). Older adults who considered the contagion risk to be very high (OR = 2.70, *p* = 0.029) were more than twice as likely to adopt this behavior compared to those who perceived being at a very low risk of transmission.

The analysis of severity perception showed that approximately one-third of participants believed COVID-19 severity was high (34.8%), or very high (31.8%). A significantly higher proportion of participants who got their information from watching television perceived COVID-19 as a very severe disease (35.8%), compared with those that did not mention television as an information source (23.6%, *p* = 0.038). Additionally, a higher proportion of those that used the web and social media perceived COVID-19 as a very severe condition (40.9%) compared to those that did not use the web (30.7%, *p* = 0.019). A higher severity perception was associated with a higher probability of staying at home, compared to those who considered the severity to be very low (high severity category OR = 2.04, *p* = 0.043; very high severity category OR = 2.82, *p* = 0.004) (Table 4).

**Figure 2:**
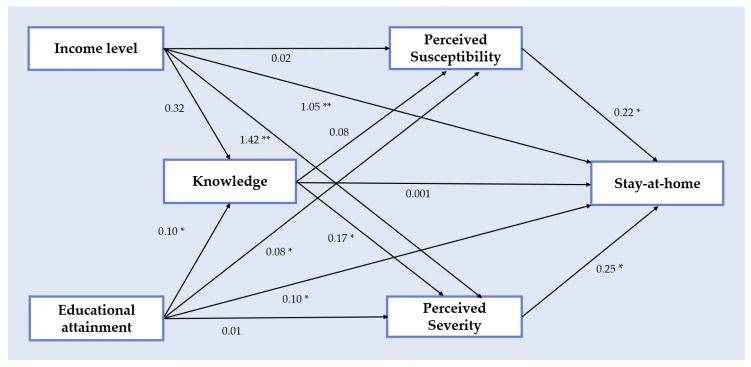
Path analysis model of stay-at-home behavior in adults (≥65 years old) in Mexico City related to income level, knowledge of coronavirus (COVID-19) symptoms, educational attainment, perceived severity of COVID-19. Coefficient of path analysis (β) * *p* < 0.05, ** *p* < 0.01. The model assumes that there is a correlation among variables at the same level.

The GSEM is depicted in Figure 2. The exogenous variables (IL and educational attainment), endogenous variables (knowledge, perceived susceptibility, and perceived severity), the outcome variable (staying at home) and the hypothetical paths are presented. The path from IL to staying at home was significant (β = 1.05, *p* < 0.001). Additionally, the IL path to perceived severity was significant (β = 1.42, *p* < 0.001), and perceived severity was positively associated with stay-at-home behavior (β = 0.25, *p* = 0.035). The path from educational attainment to stay-at-home behavior and perceived susceptibility was also significant (β = 0.10, *p* = 0.018 and β = 0.08, *p* = 0.040, respectively). Perceived susceptibility was associated with stay-at-home behavior (β = 0.22, *p* = 0.042), and educational attainment was associated with knowledge of COVID-19 symptoms (β = 0.10, *p* = 0.036). The paths connecting educational attainment, knowledge, perceived severity and stay-at-home behavior were all significant (Figure 2). However, the path from knowledge of COVID-19 symptoms to stay-at-home behavior was not significant (β = 0.001, *p* = 0.927).

Table 5 presents the total effects of IL and educational attainment on stay-at-home behavior, and the corresponding decomposition of direct and indirect effects. The total effect of IL on staying at home was β = 1.222 (*p* = 0.001), and the indirect effect (β = 0.184, *p* = 0.013) represented 15.1% of the total effect. Perceived severity was a partial mediator between IL and staying at home (IL→Perceived Severity→Stay-at-home behavior) in the GSEM. The total effects of educational attainment on stay-at-home behavior were significant when compared with the base category (incomplete elementary school), and the indirect effect (β = 0.175, *p* = 0.019) of a bachelor’s degree or higher, represented 15.0% of the total effect of this educational level on stay-at-home behavior. Perceived susceptibility was a partial mediator between educational attainment and stay-at-home behavior (Education→Perceived Susceptibility→Stay-at-home behavior) in the GSEM. Additionally, a GSEM built with a lower cut-off point for IL revealed similar results.

## 4. Discussion

This study was performed between 13 March and 20 March 2020. At the time of the study, less than 20 deaths from COVID-19 had been reported in Mexico. People had been asked by the Health Ministry to improve hygiene measures and practice physical distancing, but quarantine was not imposed; only those with respiratory symptoms were advised to stay home. Under these circumstances, 57.6% of the older adults interviewed in this study had made the decision to stay home and only go out for necessary activities. After the interviews in this study were conducted, the Mexican government moved to phase 2 of the control measures against the epidemic. A specific recommendation to stay home was given to people 60 years or older, patients with diabetes mellitus type 2 (DMT2), and those with hypertension [5]. A higher proportion of older adults staying at home since the beginning of the epidemic may have been beneficial in controlling the epidemic. In China, a higher proportion of people adopted staying-at-home behavior from the start of the epidemic: a study general population of mainland China revealed that 85% of participants stayed at home between 20 h and 24 h a day. This information was gathered using an online questionnaire administered during the first two weeks of the Wuhan outbreak [24]. A Cochrane systematic review of the effect of early quarantine due to severe acute respiratory syndrome (SARS) and Middle East respiratory syndrome (MERS) showed that 44% to 81% of new cases, and one-to two-thirds of deaths were averted [30]. Modeling studies, predicting morbidity and mortality rates, have consistently identified the benefits of quarantining a population [31]. Thus, the early implementation of quarantine, along with other public health measures, is recommend to control epidemics [30]. In countries where social distancing is difficult to achieve, higher numbers of new cases and case fatality rates have been observed [19].

Quarantine, during the virus’ rapid spread phase is advisable; however, maintaining social isolation for long periods may have negative consequences, particularly for older adults. In the present study, an 82-year-old woman stated during the interview: “I have to stay at home, and nobody can come to see me…”. For community dwelling older adults, quarantine not only means they cannot go out, but that their relatives and friends cannot come to visit them. Quarantine increases emotional distress, reduces physical activity, and decreases mental stimulation. Furthermore, social isolation increases the risk of mortality [32,33], therefore, finding an equilibrium between controlling the spread of the epidemic, and decreasing the risk of secondary negative health effects in older adults is of paramount importance [34,35].

In the present study, 53.4% of the older adults interviewed cited more frequent hand washing as a preventive behavior adopted to avoid SARS CoV-2 transmission. This measure is key to controlling community transmission of the virus, therefore, adherence to this requires improvement. Only a small proportion of the adults cited using a face mask for protection. However, at the time of the interviews, WHO had not recommended the use of face masks for the general public, which may explain the low proportion of older adults adopting this behavior.

The results of this study indicate that the COVID-19 symptom most frequently identified by older adults was fever (57.9%), followed by a cough (47.1%). In a study of the Chinese population, with a mean age of 33 years, 96.4% responded affirmatively to the question “The main clinical symptoms of COVID-19 are fever, fatigue, dry cough, and myalgia” [24]. It is difficult to compare these results with the current study group due to differences in age, and in the survey method (in this study participants had to mention the symptoms, while Chinese respondents answered “true”, “false” or “I don’t know”).

The results of the present study show that approximately 70% of the participants were aware that older adults were more likely to suffer complications from COVID-19. Older people are more likely to progress to severe disease than middle-aged adults or young people [36]. COVID-19 affects the lungs, can compromise the heart, and may produce an increase in blood glucose levels, therefore, complications such as disseminated intravascular coagulation or deep vein thrombosis are risks [36]. This is important in Mexico due to the rate of diabetes mellitus type two (DMT2), which is amongst the highest in the world [37]. Moreover, this condition makes it difficult to control a SARS CoV-2 infection. The high prevalence of obesity and hypertension in Mexico further increases the risk of mortality in COVID-19 cases [38,39,40]. On 9 July 2020, a total of 33, 526 deaths had been reported in Mexico due to SARS CoV-2 infection [19]. Older adults suffering from underlying systemic diseases are more prone to acute respiratory distress syndrome and cytokine storms [41]. Therefore, taking a multidisciplinary approach is imperative for treating these complex patients. Despite this, 30% of the older adults interviewed in this study did not perceive COVID-19 as a serious health threat, which contradicts the high mortality rate of the elderly during the epidemic. It may be that there are other threats occurring in the everyday life of older adults that make it difficult for them to perceive the severity of this disease [42].

Higher income and educational attainment were associated with a higher probability of staying at home in this study. This is consistent with other studies that have found that behaviors related to improved health are associated with socioeconomic variables in adults and older adults [43,44,45].

The classic HBM has been use in research on the adoption of preventive health behavior in several chronic and contagious diseases [46,47]. This model suggests that individuals will take action when a disease poses a serious threat and there are practices that could prevent the disease. However, the presence of barriers may make it difficult to perform preventive behavior. Furthermore, the benefits of the health behavior must be perceived as higher than the costs of adopting preventive practices [48]. This study sought to identify two components of the HBM: perceived susceptibility to, and severity of COVID-19. Our results indicate that perceptions were associated with stay-at-home behavior. Educational attainment was associated with perceived susceptibility; however, its mediation effect was relatively low. Therefore, other variables were involved in the association between education and adopting stay-at-home behavior to prevent COVID-19. Educational level has been shown to influence health behavior, and several mediation variables have been identified. For example, a follow-up study by Williams et al., demonstrated that smoking and physical activity partly mediated the association between education and the development of DMT2 [49]. Moreover, self-efficacy may mediate the effect between education and DMT2 self-management behaviors [46]. In a study of the influenza A virus subtype H [Hemagglutinin] 1 N [Neuraminidases] 1 (H1N1) epidemic in Canada, the association between hospitalization for the disease and known risk factors, such as high body mass index, smoking, and antiviral therapy could not fully mediate the association between H1N1 hospitalizations with socioeconomic variables. Therefore, other factors were involved in the relationship between social determinants and health status [50].

IL had a direct and significant effect on stay-at-home behavior in this study. There was also an indirect effect, mediated by perceived severity. However, the indirect effect was low (15.1%) and other variables may be mediating the association between IL and COVID-19 preventive behavior. Utilizing the source multiplication theory [51] informs that IL could have a multiplicative effect, as individuals have more opportunities to seek benefits, such as health services, health information, or healthier environments and family support [52]. These factors could play a role in the adoption of preventive health behaviors.

Most of the participants in our study cited television as their information source, and television in Mexico is a primary source of global information about the pandemic. In the days previous to the interviews, participants were exposed to information on the number of new COVID-19 cases in Mexico City, the closure of schools, the introduction of the DN III plan in the city, information regarding the high mortality risk for older people, and news of a patient dying from COVID-19. This type of information may affect an older person’s perceived susceptibility to, and severity of the disease, according to the belief steps of the HBM [53]. We also found that older adults in Mexico who used the web and social media for information were more likely to perceive COVID-19 as an extremely severe disease, and more likely to adopt the stay-at-home preventive behavior. How news is presented may also affect emotional responses: it appears that factual information has an effect on perceived susceptibility, while news presented in an emotional way affects perceived severity [54]. Both of these information styles are available in Mexico’s media, and the personal stories of people who died or contracted the virus are presented in TV news and talk-shows.

We found an association between educational attainment and knowledge of COVID-19 symptoms. Health information-seeking behavior has been associated with educational level, and this relationship is mediated by health literacy [55]. The association between SEP and health behavior is important, and financial security plays an essential role during outbreaks [56]. In Mexico, public health services are limited, and private facilities make important contributions to treatment delivery. However, equal treatment opportunities are jeopardized in low-income populations; this is an important factor for older adults, who have a higher disease burden than younger age groups. To facilitate access to health services for individuals with COVID-19, or those suspected of being infected, will help to control the spread of the disease.

## 5. Limitations

One of the limitations of this study is that the household income information was gathered directly from the respondent during the phone interview, which may produce information bias. Women, who were the main participants, may not be well informed about the household income. Nevertheless, it is important to consider that our study population included mostly elderly women who, in many cases, are responsible for the family budget [57]. Furthermore, a significant proportion of the participants had an education attainment above elementary school, which favors greater knowledge of family income [58,59]. Older adults who participated in the study receive a monthly pension from the government which favors them to be involved in the use of the family budget [17]. These factors may mean that information bias was limited in this study. In our model, both education and income level were associated with staying at home behavior.

The HBM has limitations, such as overemphasizing the rationality of the patients’ behavior [60]; however, it is widely used and provides a useful tool for studying health behaviors during epidemics. Furthermore, it is considered a good theory to include when modeling epidemics [61]. While the main components of the HBM were analyzed in this study, self-efficacy and cues to action were not investigated. Therefore, the role of the self-efficacy dimension in the adoption of health preventive behaviors, in the context of an epidemic, requires further study in older adults. Another limitation of the study concerns the small number of males in the sample, which precluded a sex-specific analysis. The representativeness of the population was limited as the sample was conducted on older people living in the south-eastern area of the city, which primarily houses middle and low economic groups. It is likely that people at other economic levels, such as high or very low incomes, would have different responses to the epidemic. However, the socioeconomic status of the respondents is similar to a large portion of older adults in Mexico City. The literature search for this study was completed on 14 August 2020; some of the information presented here could be subject to change as more knowledge is acquired regarding this pandemic in more recent studies.

Different types of bias may had occurred in this study as well. For instance, social desirability bias, which occurs when the participants answer with socially desirable rather than truthful answers. To reduce this risk, the interviewers explained to the participants that their answers would remain confidential and their names would not be link to their answers.

Follow-up studies may be useful to identify factors involved in the decision-making process leading to the adoption of preventive health behaviors during epidemics, and may contribute to the identification of public health strategies for the early control of epidemics. Timely intervention is particularly important in epidemics with a high R_0_ due to the exponential growth of new cases and the difficulties faced by different countries to control the epidemic.

## 6. Conclusions

More than one-third of the older adults in this study had decided to not adopt stay-at-home preventive behavior. Those with a lower educational attainment and lower IL were less likely to stay at home. Higher perceived susceptibility and severity were associated with staying at home as precautionary measure against COVID-19. This information is essential to identify target groups for public health interventions aiming to control this pandemic, which has caused an exceptionally large number of deaths in the world population, and for which subsequent waves of infection need to be controlled.

## Figures and Tables

**Table 1:** Telephone interview questionnaire for adults aged 65 years and older, on the coronavirus (COVID-19) pandemic in Mexico City.

1. Compared to other people, how likely are you to get COVID-19?
(a) Very high
(b) High
(c) Low
(d) Very low
2. How severe do you think COVID-19 infection is?
(a) Not at all serious
(b) Slightly serious
(c) Moderately serious
(d) Severely serious
3. Do you know what the main symptoms of this infection are?
(a) No
(b) Yes, mention them: ____
4. Which age group has the greatest chance of complications if they get COVID-19?
(a) Children
(b) Young adult
(c) Adults
(d) Older adults
5. Have you taken any steps to prevent COVID-19 contagion?
(a) No
(b) Yes, mention them: ____
6. What has been your main source of COVID-19 information?
(a) Television
(b) Radio
(c) Newspaper
(d) Family and friends
(e) Web/social media
(f) Other_____

**Table 2:** Sociodemographic characteristics, knowledge, and sources of information on the coronavirus (COVID-19) pandemic in 380 adults (≥65 years old) in Mexico City. Number of respondents (*n*) and % response.

Characteristic		
Age mean (± sd *)	72.9	(±8.0)
	*n*	(%)
Sex		
Men	91	23.9
Women	289	76.1
IL **		
Low	168	44.2
Middle	212	55.8
Years of schooling		
<3	74	19.5
3–6	58	15.3
7–8	83	21.8
9–10	69	18.1
>10	96	25.3
Symptoms of COVID-19		
Fever	220	57.9
Cough	179	47.1
Tiredness	41	10.8
Breathing difficulties	125	32.9
Flue/sore throat	131	34.5
Headache	107	28.2
Other	87	22.9
Did not know any of the symptoms	46	12.1
Age group at highest risk of COVID-19 complications		
Children	22	5.8
Young adults	2	0.5
Middle-aged adults	18	4.7
Older adults	264	69.5
All age groups equally	74	19.5
Sources of information on COVID-19		
Television	257	67.6
Radio	115	30.3
Newspaper/magazines	53	13.9
Web/Social-Media	44	11.6
Family/friends	60	15.8

* sd = standard deviation,** IL = Income level.

**Table 3:** Odds ratios of stay-at-home behavior and sociodemographic characteristics, knowledge and sources of information of the COVID-19 pandemic for adults (≥65 years old) in Mexico City.

	Stay-at- Home No	Stay-at- Home Yes	OR	(95% CI)	*p*-Value
Characteristic					
Age mean (± sd *)	72.8 (±7.8)	73.0 (±8.1)	1.00	(0.97, 1.03)	0.875
	*n* (%)	*n* (%)			
Sex					
Men	45 (49.5)	46 (50.6)	1 (Reference) **		
Women	132 (45.7)	157 (54.3)	1.16	(0.73, 1.86)	0.529
IL ***					
Low	106 (63.1)	62 (36.9)	1 (Reference) **		
Middle	71 (33.5)	141 (66.5)	3.40	(2.22, 5.19)	<0.001
Years of schooling					
<3	50 (67.6)	24 (32.4)	1 (Reference) **		
3–6	21 (36.2)	37 (63.8)	3.67	(1.78, 7.57)	<0.001
7–8	44 (53.0)	39 (47.0)	1.85	(0.96, 3.54)	0.064
9–10	24 (34.8)	45 (65.2)	3.91	(1.95, 7.82)	<0.001
>10	38 (39.6)	58 (60.4)	3.18	(1.68, 6.01)	<0.001
Symptoms of COVID-19					
Fever ^a^	90 (40.9)	130 (59.1)	1.72	(1.14, 2.60)	0.010
Cough ^b^	76 (42.5)	103 (57.5)	1.37	(0.91, 2.05)	0.129
Tiredness ^c^	19 (46.3)	22 (53.7)	1.01	(0.53, 1.94)	0.974
Breathing difficulty ^d^	60 (48.0)	65 (52.0)	0.92	(0.60, 1.41)	0.697
Flue/sore throat ^e^	65 (49.6)	66 (50.4)	0.83	(0.54,1.27)	0.389
Headache ^f^	46 (43.0)	61 (57.0)	1.22	(0.78, 1.92)	0.380
Did not know any of the symptoms ^g^	25 (54.4)	21 (45.7)	0.70	(0.38, 1.30)	0.262
Age group with most COVID-19 complications					
Other age groups	63 (54.3)	53 (45.7)	1 (Reference) **		
Old adults	114 (43.2)	150 (56.8)	1.56	(1.01, 2.43)	0.046
Sources of information on COVID-19					
Television	114 (44.4)	143 (55.6)	1.32	(0.86, 2.03)	0.210
Radio	70 (60.9)	45 (39.1)	0.43	(0.28, 0.68)	<0.001
Newspaper/magazines	27 (50.9)	26 (49.1)	0.82	(0.46, 1.46)	0.493
Web/Social media	10 (22.7)	34 (77.3)	3.36	(1.61, 7.02)	<0.001
Family/friends	27 (45.0)	33 (55.0)	1.08	(0.62, 1.88)	0.789

** 1 (Reference): Reference category for odds ratio calculation; Reference category for each symptom: not mentioning fever ^a^, not mentioning cough ^b^, not mentioning tiredness ^c^, not mentioning breathing difficulties ^d^, not mentioning flue/sore throat ^e^, not mentioning headache ^f^, not mentioning any of the above symptoms ^g^; * sd = standard deviation, *** IL = Income level, OR = odds ratio, 95% CI = 95% confidence intervals.

**Table 4:** Distribution and odds ratios of the preventive behavior “stay-at-home”, associated with the perception of becoming infected with Severe Acute Respiratory Syndrome Coronavirus 2 (SARS-CoV-2) and severity of the disease in adults (≥65 years old) in Mexico City.

Perception	Stay-at- Home No *n* (Row %)	Stay-at- Home Yes *n* (Row %)	Total *n* (Column %)	OR	(95% CI)	*p*-Value
Perceived Susceptibility						
Very low	25 (61.0)	16 (39.0)	41 (10.8)	1 (Reference) *		
Low	79(47.9)	86(52.1)	165 (43.4)	1.70	(0.85, 3.42)	0.136
High	58 (43.6)	75 (56.4)	133 (35.0)	2.02	(0.99, 4.13)	0.054
Very High	15 (36.6)	26 (63.4)	41 (10.8)	2.70	(1.11, 6.62)	0.029
Perceived Severity						
Very low	27 (60.0)	18 (40.0)	45 (11.8)	1 (Reference) *		
Low	52 (63.4)	30 (36.6)	82 (21.6)	0.87	(0.41, 1.83)	0.704
High	56 (42.4)	76 (57.6)	132 (34.8)	2.04	(1.02, 4.05)	0.043
Very High	42 (34.7)	79 (65.3)	121 (31.8)	2.82	(1.40, 5.71)	0.004

OR = odds ratio, 95% CI = 95% confidence intervals, * 1 (Reference): Reference category for odds ratio calculation.

**Table 5:** Results of a mediation analysis of the stay-at-home COVID-19 preventive behavior, and the total effects, indirect effects and direct effects of income level and educational level in adults (≥65 years old) in Mexico City.

**Variable**	**β Coefficient**	**(95% CI) ^1^**	***p*-Value**
Income Level			
	Total		
Middle vs. Low	1.222	(0.813, 1.632)	0.001
	Indirect		
	0.184	(0.040, 0.329)	0.013
	Direct		
	1.038	(0.600, 1.476)	0.001
**Education level (years of schooling)**	**β Coefficient**	**(95% CI) ^1^**	***p*-Value**
	Total		
3–6 vs. <3	1.300	(0.553, 2.048)	0.001
	Indirect		
	0.161	(−0.043, 0.365)	0.122
	Direct		
	1.300	(0.553, 2.048)	0.001
	Total		
7–8 vs. <3	0.613	(0.091, 1.136)	0.021
	Indirect		
	0.061	(−0.112, 0.233)	0.491
	Direct		
	0.553	(0.024, 1.081)	0.040
	Total		
9–10 vs. <3	1.363	(0.621, 2.105)	0.001
	Indirect		
	0.131	(−0.040, 0.302)	0.132
	Direct		
	1.231	(0.501, 1.961)	0.001
	Total		
>10 vs. <3	1.157	(0.461, 1.852)	0.001
	Indirect		
	0.175	(0.029, 0.321)	0.019
	Direct		
	0.982	(0.298, 1.675)	0.005

^1^ Boot strap method 1000 replications, 95% CI = 95% confidence intervals.
